# Ulcerative Colitis Narrative Global Survey Findings: Communication Gaps and Agreements Between Patients and Physicians

**DOI:** 10.1093/ibd/izaa257

**Published:** 2020-10-15

**Authors:** David T Rubin, Ailsa Hart, Remo Panaccione, Alessandro Armuzzi, Ulla Suvanto, J Jasper Deuring, John Woolcott, Joseph C Cappelleri, Kathy Steinberg, Laura Wingate, Stefan Schreiber

**Affiliations:** 1 University of Chicago Medicine, Inflammatory Bowel Disease Center, Chicago, Illinois, USA; 2 IBD Unit, St. Mark’s Hospital, London, UK; 3 Division of Gastroenterology and Hepatology, Department of Medicine, University of Calgary, Calgary, Alberta, Canada; 4 IBD Unit, Fondazione Policlinico Universitario A. Gemelli IRCCS–Università Cattolica del Sacro Cuore, Rome, Italy; 5 Crohn and Colitis Association of Finland, Tampere, Finland; 6 Pfizer Inc, Rotterdam, The Netherlands; 7 Pfizer Inc, Collegeville, Pennsylvania, USA; 8 Pfizer Inc, Groton, Connecticut, USA; 9 The Harris Poll, New York, New York, USA; 10 Crohn’s & Colitis Foundation, New York, New York, USA; 11 Department of Internal Medicine, University Hospital Schleswig-Holstein, Kiel, Germany

**Keywords:** inflammatory bowel disease, physician-patient relationships, quality of care, shared decision-making, survey

## Abstract

**Background:**

The Ulcerative Colitis (UC) Narrative global surveys examined patient and physician perspectives on living with UC and tried to identify gaps in optimal care. Questions explored patient-physician interactions, UC management goals, and resources for improving communication.

**Methods:**

Questionnaires were conducted across 10 countries, covering aspects of UC including diagnosis, treatment, and impact on patient quality of life, in addition to standard demographic information. Descriptive statistics were calculated.

**Results:**

Globally, 2100 patients and 1254 physicians were surveyed (from August 2017 to February 2018). Results showed 85% of patients were satisfied with the communication they had with their physician, including discussions relating to symptoms (86%) and medication options (81%). However, 72% of patients wished for more information and support at initial diagnosis, and 48% did not feel comfortable talking to their physician about emotional concerns. Most patients (71%) set UC management goals with their physician. Both patients (63%) and physicians (79%) wished for longer appointments. Although 84% of physicians believed patient advocacy organizations to be important in UC management, more than half (54%) never discussed them with patients.

**Conclusions:**

These survey results highlight overall patient satisfaction with patient-physician communication but emphasize areas for improvement, such as patient desire to have more information earlier in their disease course. There is an unmet need for better information, materials, and support. Physicians need to consider which of the available tools and resources can help patients talk more openly, and accurately, because informed patients are more likely to engage with physicians in a shared decision-making process.

## Graphical Abstract

**Graphical Abstract:**
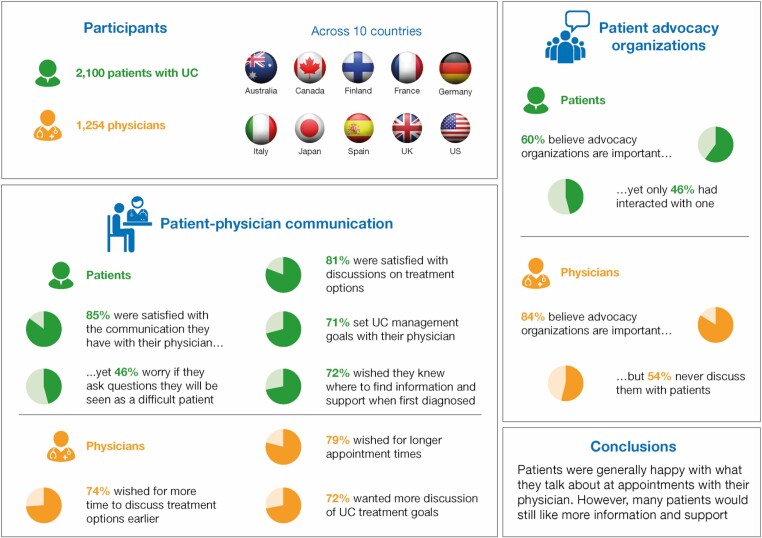

## INTRODUCTION

Ulcerative colitis (UC) is a chronic inflammatory bowel disease (IBD) that affects the colon and rectum, and the extent of mucosal involvement varies from proctitis to pancolitis.^1, 2^ Most often, UC is diagnosed in early adulthood and is clinically characterized by relapsing and remitting symptoms including diarrhea, rectal bleeding, abdominal pain, fecal urgency, weight loss, and fatigue.^1, 2^ More than one-third (37%) of patients with UC report long-term chronic intermittent symptoms, and a proportion will progress to chronic active disease and not enter remission.^3^ Because of the chronic nature of UC and the significant impact of disease on quality of life,^4, 5^ the relationship between patients and their physician is important in understanding and implementing appropriate management strategies.^6-8^

When patients and clinicians engage in shared decision-making, it can lead to increased patient satisfaction and improved outcomes.^9^ A critical step in the shared decision-making process is the communication between patients and physicians,^7^ and there is a need for comprehensive patient-physician communication to cover the range of medical and surgical treatment options.^10^ Patient perceptions and opinions of different treatment options need to be integrated into treatment decisions. However, patients’ and physicians’ perceptions and opinions regarding the management of UC can differ.^11^ A previous survey of patients with UC and health care professionals across Western Europe and Canada revealed that health care professionals may fail to recognize the issues that are important to patients.^12^

The UC Narrative was composed of 2 related global surveys that separately examined the perspectives of patients and physicians across a range of countries. Although most IBD-related surveys encompass patients with UC and patients with Crohn disease,^13-15^ these surveys were specific to UC. The UC Narrative survey questions explored aspects of living with UC, including day-to-day disease impact, disease management, goal-setting, and communication between patients and physicians. Here, we present patient and physician responses to questions relating to communication during the management of UC. The hope is that identifying gaps will lead to ways to enhance communication and improve patient-physician interactions, helping patients to cope with symptoms and increase the likelihood that they will adhere to treatment. Improving patient-physician communication helps patients make more informed decisions about medications and surgical options, thereby making them an active partner in their disease management.^7, 9, 10^

## MATERIALS AND METHODS

### Study Design and Populations

The UC Narrative is a collaborative global initiative sponsored by Pfizer Inc that includes direction from an advisory panel comprising adults living with UC, gastroenterologists, IBD nurses, a psychologist, and representatives of IBD patient advocacy organizations from 10 countries: Australia, Canada, Finland, France, Germany, Italy, Japan, Spain, the United Kingdom, and the United States. The goal of the UC Narrative is to improve outcomes for people living with UC internationally by identifying common and country-specific barriers to better care and propose solutions to overcome these barriers. The initiative involved 2 related global surveys, one patient-based and one physician-based. Survey topics were sometimes addressed in multiple ways, or by asking a few slightly different questions, recognizing that responses may vary based on how each individual question was asked. Full details of the number of patients and physicians in each country who completed the survey can be found in Supplementary Table 1.

Patient- and physician-based surveys were conducted between August 2017 and February 2018 by The Harris Poll. The patient and physician questionnaires can be found in Supplementary Materials 1 and 2, respectively. The surveys were designed to assess multiple aspects of UC and its management in addition to standard demographic information (Table 1). The physician questionnaire mirrored the patient questionnaire where applicable. Physicians were asked to base their survey responses on their experiences of treating patients with moderate to severe UC (defined as those who had ever taken more prescription medications than just 5-aminosalicylates for their disease). Patients were recruited from databases (held by The Harris Poll and partners) of individuals who agreed to participate in market research studies after being recruited through a variety of both online (eg, social media, targeted banner ads) and offline (eg, magazine ads, targeted phone recruitment) sources. Full details of patient recruitment can be found in Supplementary Materials 3.

**TABLE 1.** Key Areas Covered by the UC Narrative Global Surveys

Key Areas of Patient and Physician Surveys*	Examples of Topics Covered by Survey Questions
Demographics^†^	Age, country of residence, sex
Diagnosis of UC	Age at diagnosis, time between symptom onset and diagnosis
UC symptoms	Overall health, symptoms experienced, daily bathroom visits, remission status (as considered by patients), number of flares (in the past 12 months)
Impact of UC	Emotional impacts of UC, family impacts of UC, work impacts of UC, missed events because of UC, top worries because of UC
Management of UC^†^	Important issues in management of UC, goal-setting (treatment and day-to-day), priorities for routine appointments
Treatment	Medication (ever taken, current), satisfaction with current medication, medication choices, hospital visits in the past 12 months (as reported by patients)
Patient-physician communication^†^	Satisfaction with communication, topics that patients felt physicians could better understand
Knowledge of UC	Disease knowledge, treatment knowledge
Information and support^†^	Patient advocacy organizations, tools to improve patient-physician relationships

*Where applicable and appropriate, the physician questionnaire mirrored the patient questionnaire.

^†^Area covered in this article.

Patients were ≥ age 18 years and self-reported that they had received an endoscopic confirmed diagnosis of UC, had not had a colectomy, had visited a gastroenterologist/internist in the past 12 months, and had ever taken prescription medication for their UC. Disease severity in this study was defined using a novel patient-reported medication history. Patients with moderate to severe UC were defined as those who had ever taken an immunosuppressant or a biologic for their UC or had taken corticosteroids for ≥4 of the past 12 months. Patients with mild UC were defined as those who had never taken a biologic or immunosuppressant and those who had taken corticosteroids for ≤3 months of the past 12 months. Patients who had never taken a prescription medication for their UC or had only ever taken 5-aminosalicylates were excluded. Patients with mild UC were capped at 20% of total survey respondents to focus the survey on patients with moderate to severe UC.

Care for UC may be provided by different specialties in different countries. Eligible physicians across all 10 countries must have practiced as a gastroenterologist, an internist with a gastroenterology focus, a gastroenterologist internist, or in gastroenterology surgery (full details of physician recruitment can be found in Supplementary Materials 3). To meet inclusion criteria, physicians were required to see ≥10 patients with UC per month (≥5 in Japan), and at least 10% of their current patients had to be taking a biologic to manage their UC. In the United States, physicians had to be licensed in the state they practiced, not practice in Vermont (to comply with the 2009 Physician Payments Sunshine Act),^16^ and not be associated with Kaiser Permanente (a standard requirement for Pfizer-funded research).

Questions on both the patient and physician questionnaires required respondents to provide a numeric response, to select a single option or multiple options from a list, or to indicate their level of agreement with a statement (from “strongly disagree” to “strongly agree”).

### Analyses of Patient and Physician Surveys

Survey responses were analyzed globally and by country. Descriptive statistics were used to assess patient and physician responses. Analyses were primarily conducted in IBM SPSS.^17^ The raw data were analyzed by The Harris Poll. In the United States, physician results were weighted by region, years in practice, and physician sex. In other countries, physician results were weighted by age and sex to ensure alignment with the actual proportion in the population of gastroenterologists in each country.^18^ For the global patient and physician data, a postweight was applied to adjust for the relative size of each country’s adult population within the total adult population (≥ age 18 years) across all countries surveyed.^18^ The unweighted sample sizes reflected the total number of patients who completed the survey in each country, and all reported percentages were calculated based on the weighted global total described here.

Individual country results were combined into a “global total” such that each country carried a weight that was proportionate to its population within the global total of countries surveyed. Proportionate weighting for combining multicountry data into a single total is a common practice because it relies on externally recognized population data (this survey used the International Data Base of the U.S. Census Bureau) to achieve a global total that more accurately represents the real-world relative to the adult populations surveyed. The prevalence of IBD in each country was not included in the weighting.

## ETHICAL CONSIDERATIONS

In the United States, the research method and survey questionnaire were reviewed and received institutional review board approval (Western Institutional Review Board protocol number 20171627). The surveys were noninterventional, were not intended to provide clinical data for treatment decisions, and were not conducted as a clinical trial for any endpoints; ethics approval was therefore not required. All respondents provided their informed consent and were compensated on behalf of the investigators by the sponsor (Pfizer Inc) for their participation in the survey.

## RESULTS

### Survey Respondents

Across the 10 countries, 2100 patients with UC completed the patient survey and 1254 physicians responded to the physician survey. The mean patient age was 40.8 years (SD = 12.4; median = 38); 53% of patients were male; based on medication history, 82% had moderate to severe UC and 67% described their UC as being in remission (defined as controlled with few to no symptoms). Overall, 37%, 48%, and 15% of patients described their current overall health as good/excellent, fair, or poor, respectively. At the time of the survey, patients reported that a mean of 8.8 years (SD = 9.2; median = 5) had passed since their UC diagnosis.

Globally, physicians had a mean age of 47.6 years (SD = 10.0), had been in their specialty practice for a mean of 16.4 years (SD = 8.4; median = 15) years, and reported seeing a mean of 39.8 patients (SD = 37.2; median = 30) with UC per month; 85% were male. In total, 36% of physicians described their medical practice as mostly office- or clinic-based, 23% were mostly hospital- or lab-based, 23% were equally split between being hospital-based and office/clinic-based, and 18% were exclusively hospital- or lab-based.

### Patient-Physician Interactions

**FIGURE 1.**
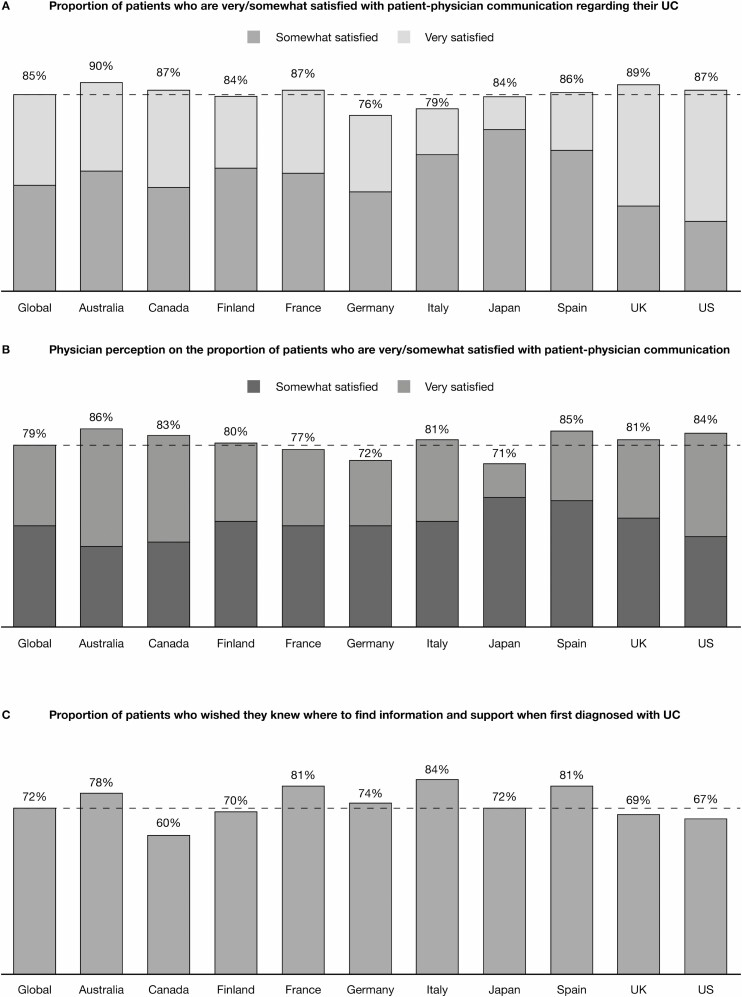
Patient and physician views on communication. Proportional weighting was used to generate global totals. Dashed lines indicate the global total.

Worldwide, the majority of patients (85%) were satisfied with the communication they had with their physician regarding their UC (Fig. 1A). This result was in agreement with physician perceptions on the proportion of patients who were satisfied with patient-physician communication; they estimated that 80% of their patients were satisfied (Fig. 1B). Globally, a high proportion of patients were satisfied with the discussions they had with their physician about their symptoms (86%) and about medication options, including benefits and side effects (81%). Although patient satisfaction was high overall, patients wished they had been better informed earlier after diagnosis. The majority of patients (72%) wished that they had known where to find information and support at initial diagnosis (Fig. 1C), and more than half (54%) of patients wished that their physician had discussed all available treatment options earlier.

Globally, physicians recognized the importance of effective communication with their patients, with most physicians (74%) taking steps to improve their communication skills. Most physicians (71%) wished there was a way that their patients could communicate with them more frequently while experiencing symptoms between visits. Physicians also noted the importance of communicating with patients soon after diagnosis; almost three-quarters (74%) wished for more time to discuss treatment options earlier so that their patients had a better idea of their choices. The majority of physicians (87%) recognized that patients with UC who were involved in making treatment decisions tended to be more satisfied with their treatment experience than those who were not as involved (ranging from 76% in Japan to 96% in Canada).

Almost half of patients (48%) said that they did not feel comfortable talking to their physician about emotional concerns. A similar proportion of patients (55%) did not feel comfortable talking to their physician about their sex life and personal relationship concerns. Approximately one-third of patients (35%) said that they wished their physician better understood how much their UC impacted their quality of life. Only half of physicians (49%) said that they discussed the impact of UC on mental or emotional health with their patients.

### Routine Appointments

**FIGURE 2.**
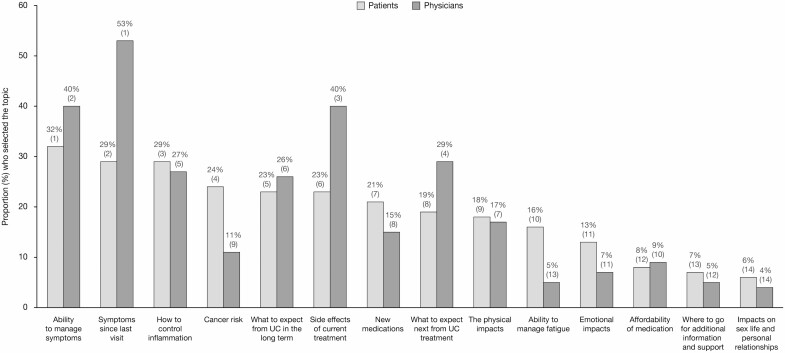
Global patient and physician priorities for routine appointments. Patients and physicians could choose up to 3 options. The number in parentheses above each bar represents the overall patient or physician ranking for each response. Affordability of medication was not requested in France, Germany, Italy, Japan, Spain, or the United Kingdom.

**FIGURE 3.**
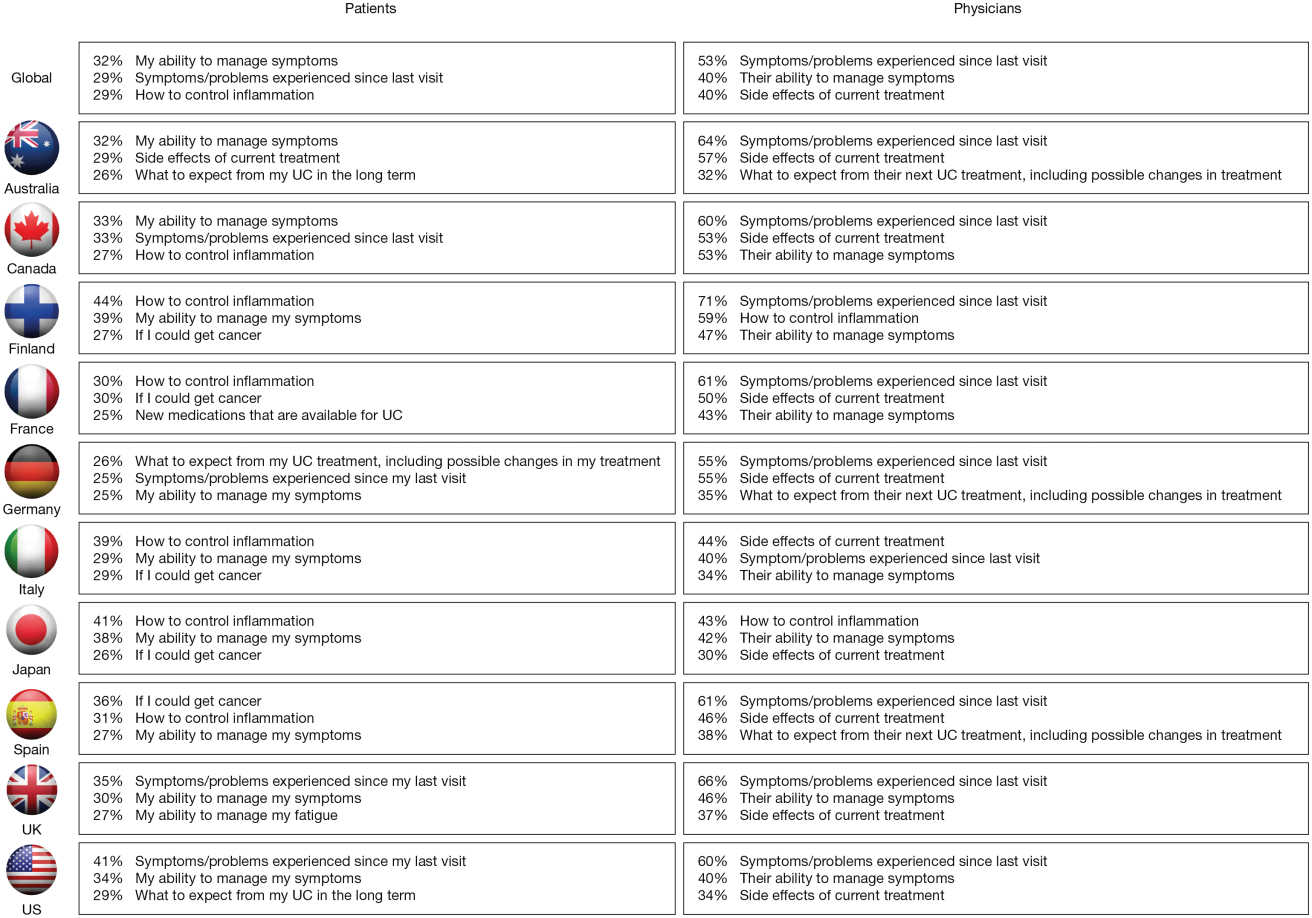
The top 3 patient and physician priorities for routine appointments, by country.

Worldwide, survey responses showed some overlap between patient and physician responses in their priorities for discussion at routine appointments (Fig. 2). The top priorities selected by patients were “ability to manage symptoms” (32%), “symptoms/problems experienced since last visit” (29%), “how to control inflammation” (29%), and “cancer risk” (24%). Physicians’ highest priorities included “symptoms since last visit” (53%), “ability to manage symptoms” (40%), and “side effects of current treatment” (40%) (Fig. 2). The greatest discordance between patient and physician prioritization was observed for symptoms/problems since last visit (selected by 29% of patients and 53% of physicians), cancer risk (selected by 24% of patients and 11% of physicians), and side effects of current treatment (selected by 23% of patients and 40% of physicians). On a country level, the greatest divergence between patients’ and physicians’ top 3 priorities for routine appointments was noted in France and Spain (Fig. 3).

Worldwide, 89% of patients stated that they were honest with their physician when discussing their experiences with UC. When physicians were asked about patient honesty, 84% agreed that their patients were honest with them, although the majority (65%) only somewhat agreed. Most patients (81%) felt comfortable raising concerns and fears with their physician. However, even those patients who felt comfortable still reported some unspoken apprehension; approximately half (49%) of patients often regretted not telling their physician more during visits, and 57% wished they talked more about their fears of medical treatments. Similarly, almost half of patients (46%) worried that if they asked too many questions then their physician would see them as a difficult patient, which would affect the quality of care they received. Globally, almost two-thirds (63%) of patients wished they had more time at appointments; this desire ranged from 52% in Canada to 76% in Italy. Globally, 47% of patients agreed that their physician rarely had time to address all their questions and concerns. A strong majority (79%) of physicians also wished that they had more time at appointments, ranging from 70% in Germany to 91% in Spain.

### Goals for UC Management

**FIGURE 4.**
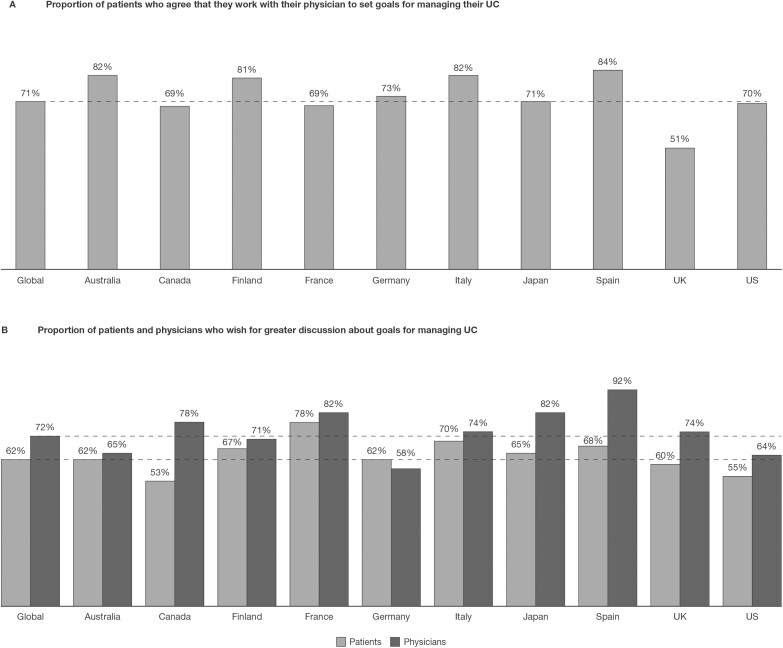
Patient-physician perceptions on goals for UC management. Proportional weighting was used to generate global totals. Dashed lines indicate the global total.

Globally, the majority (71%) of patients agreed that they currently worked with their physicians to set goals for managing their UC, ranging from 51% in the United Kingdom to 84% in Spain (Fig. 4A). Patients (62%) and physicians (72%) alike expressed a desire for greater discussion of UC treatment goals, although responses varied by country (Fig. 4B). Patients reported high levels of satisfaction with discussions on how current medications might help them reach their long-term treatment goals (81%) or day-to-day goals (80%). However, only 59% of physicians reported ever having discussed lifestyle goals (eg, the ability to participate in a hobby or be able to travel) with their patients as part of their UC management. The proportion of physicians who discussed lifestyle goals on a regular basis was lower (26%), and 15% of physicians discussed lifestyle goals only when asked by their patients.

### Resources for Improving Relationships With Patients and the Role of Patient Advocacy Organizations

Physicians were asked their opinion on which resources would help them improve their relationships with patients (Table 2). Globally, the top resources that physicians felt could help improve their patient relationships were an online tool or smartphone application for patients to better monitor and track their activities and symptoms (42%), advice for patients on where to get reliable information to help them manage their disease (39%), longer visits (39%), and the ability to refer patients to chronic disease management classes (38%).

**TABLE 2.** Resources to Help Improve Patient Relationships, as Selected by Physicians

Resources	Global Total* (%)	Range^†^ (%)	Median^†^ (%)
An online tool or smartphone application to better monitor and track patient activities and symptoms	42	27-70	43
Advice on where to get reliable information to help patients manage their disease	39	29-71	39
Longer visits	39	18-60	40.5
The ability to refer patients to chronic disease management classes	38	18-59	39
Informed resources to provide to patients	37	18-64	33.5
More information about UC in general to give to patients	36	24-63	36.5
A list of other health care professionals in my area (eg, psychologists, nutritionists, IBD nurses, rheumatologists, dermatologists) to refer to patients to aid in UC treatment	35	18-59	34.5
More information about UC treatment options available to give to patients	35	18-62	34
Clarity on patients’ personal treatment goals and whether they are meeting them	33	22-59	39
More tools to help patients prepare for physician visits (eg, list of questions to ask, informative brochures)	33	12-53	31.5
A tool explaining mechanics of UC	31	18-53	32.5
Discussion of whether patients take their medication(s) exactly as prescribed	28	15-51	29
More frequent visits	28	12-38	25.5
An IBD nurse to help with management (among patients without IBD nurses)	21	0-39	15
Other methods of communication (eg, telephone, video conversations, email)	20	7-42	19
Better access to colonoscopies	16	8-32	18
Other	1	0-5	1
Nothing would help improve patient relationships	1	0-6	1.5

Physicians were asked, “In thinking about your patients with moderate to severe UC, which of the following, if any, would help improve your patient relationships?” Physicians could select any that applied from a list of 18 possible options (including “other” and “nothing”).

*Proportional weighting was used to generate global totals.

^†^Range and median values were calculated from individual country data.

**FIGURE 5.**
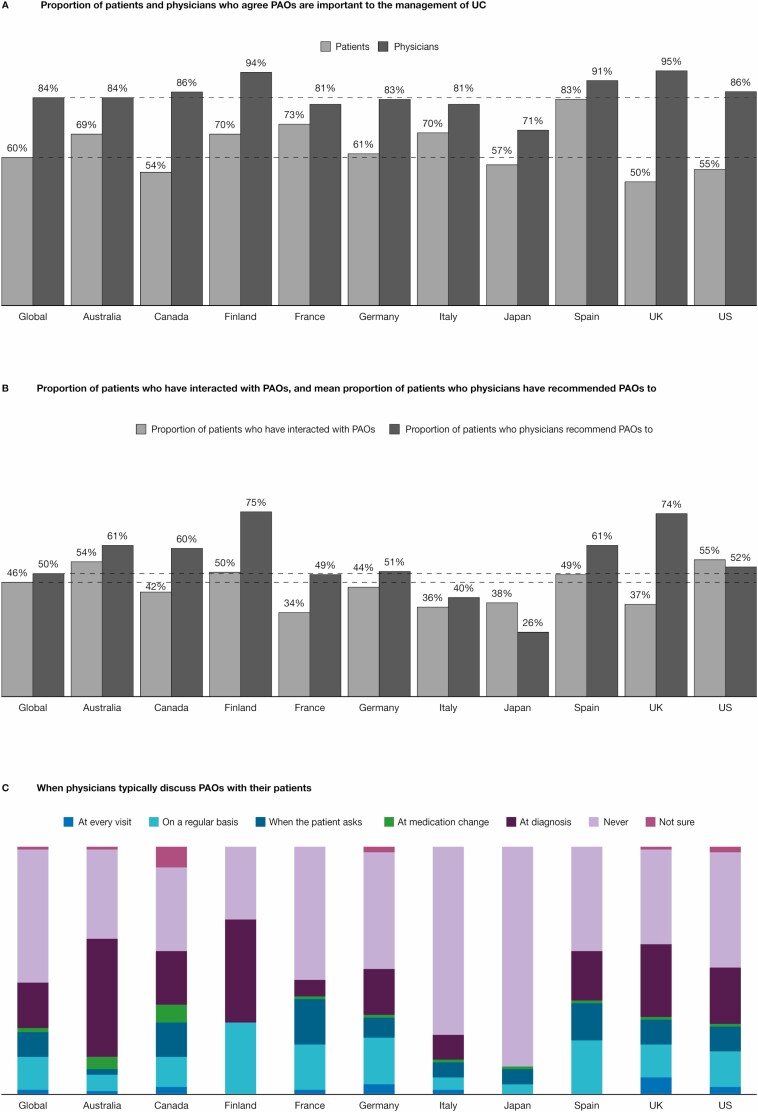
Patient-physician communication on the importance of PAOs and the role of PAOs in the management of UC. Proportional weighting was used to generate global totals. Dashed lines indicate the global total. PAO indicates patient advocacy organization.

Patient advocacy organizations have been identified as a source of reliable information and support for patients. Worldwide, the majority (84%) of physicians believed patient advocacy organizations to be important in their patients’ management of UC, although views differed by country, with 71% of physicians in Japan agreeing compared with 95% in the United Kingdom (Fig. 5A). Despite this recognition of the role of these organizations, physicians only recommended them as a source of information or support to a mean of 49.5% of their patients (Fig. 5B). Consistent with this result, approximately half (54%) of physicians said that they never discussed patient advocacy organizations with their patients (Fig. 5C). Worldwide, 18% of physicians only discussed patient advocacy organizations with their patients at diagnosis or initial consultation, and only 13% discussed them on a regular basis as part of disease management (Fig. 5C).

Worldwide, 60% of patients said that patient advocacy organizations were important for the management of their UC (Fig. 5A). However, less than half (46%) of patients said that they had ever interacted with a patient advocacy organization (Fig. 5B), and less than a quarter of patients answered yes when asked if they had reached out to a patient advocacy organization (23%) or relied on information from a patient advocacy organization or support group to help make choices about treatment or disease management (22%). Overall, 42% of patients felt that their physician could have better explained how to access information and support from patient advocacy organizations. Of those patients who had interacted with patient advocacy organizations, 72% wished that they had known about them earlier. The patient survey also revealed that 37% of patients had participated in a support group. In total, 15% of patients had participated in support groups that met in person and 27% had participated in online support groups.

## DISCUSSION

We have reported findings from 2 complementary surveys developed by the Global UC Narrative Advisory Panel. The surveys explored themes relating to patient-physician interactions, UC treatment and treatment goals, and management of UC, with the aim of better understanding the perspectives of both patients and physicians about aspects of living with UC. The patient and physician surveys were conducted independently of each other, with no direct link between responses. One strength of these surveys was that they were conducted across 10 countries, allowing data to be individualized by country or to be pooled to provide a global perspective of differences between patient and physician views and attitudes.

The importance of communication in patient decision-making for UC has been highlighted in other studies.^6, 7, 10, 19^ Our findings show that physicians and patients were generally in agreement on the topics they prioritized for discussion during routine appointments. Consistent with previous surveys,^20, 21^ symptom control was a high priority. However, the views of patients and physicians are not always aligned—patient and physician perspectives on remission have been shown to vary.^6, 12^ This discrepancy may lead to different perceptions of treatment goals, highlighting a need for better communication, which can lead to greater adherence to treatment.^22^ The findings of the current survey show that the majority of patients with UC worldwide were satisfied with how they communicated with their physician about their UC.

However, these surveys also highlighted areas where changes can be made to enhance patient-physician interaction. More time should be allocated to discuss UC and treatment options earlier during the course of the disease. Empowering patients through information and support facilitates shared decision-making and a treat-to-target approach, in which physicians and patients are partners.^7, 10, 23^ The overall goal of this approach is to educate patients so that they are informed about their treatment options, have confidence in how their disease is managed, and are therefore more likely to adhere to the chosen therapy.^23^

Both patients and physicians recognize the importance of treatment goals, with many patients identifying concerns important to them for managing their UC. Patients were satisfied with discussions on how medications can help them reach long-term treatment goals. However, not all physicians discussed lifestyle goals with their patients, and only one-quarter discussed them on a regular basis. This finding is in agreement with a Swiss study that found that although patient and physician perceptions on IBD treatments aligned, physicians focused on long-term objective goals whereas patients focused on shorter-term measures such as stress management, nutritional advice, and information on treatment effects.^24^ There remains an unmet need for more detailed discussion of goals between patients and physicians, using appropriate language to aid patient understanding, so that the views of both parties are clearly understood, which will enable greater alignment.

The current survey showed discordance on prioritization for some topics, including cancer risk, which patients viewed as a higher priority than physicians, a finding that is consistent with an American survey.^19^ Whereas physicians’ perspectives of cancer risk likely relate to the rarity of cancer, patients’ perspectives may reflect the subjectivity of fear. Nearly half of the patients surveyed here were not comfortable discussing emotional concerns with their physician. This finding is in agreement with previously reported patient-physician surveys, which found that patients rarely described the emotional impacts of their disease with their physician^6^ and that patients felt that there was not enough emotional and psychological support.^25^ Online forums can provide access to the emotional support that many patients feel is lacking when discussing UC with their physician.^26, 27^ However, most patients express doubts about the quality of IBD information posted on social media or the internet,^28, 29^ which further highlights the need for increased use of patient advocacy organizations.

Several studies have reported that physicians or other health care providers are the primary source of information for patients,^12, 30–32^ although patient advocacy organizations can also be a reliable source of information. In this survey, less than one-quarter of patients said they had reached out to a patient advocacy organization or used a patient advocacy organization as a source of information. Similar results were obtained from a Spanish survey^31^ and a survey of European and Israeli patients.^32^ Despite physicians recognizing the importance of patient advocacy organizations, many never discuss them with their patients. Notably, the majority of patients who had interacted with a patient advocacy organization wished they had done so earlier, making this an important discussion topic.

In this global survey, a strong majority of physicians wished that they could spend longer with their patients, and almost three-quarters wished for more time to discuss treatment options with patients soon after diagnosis. In a survey conducted by the IBD2020 global forum, 2 of the factors significantly associated with perceived excellent or very good quality of care were consultation length and the quality of specialist communication.^13^ It has also been suggested that there is an unmet need for tools to aid discussion and align treatment goals.^6^ In this global survey, the number one resource that physicians felt could most improve relationships with patients was an online tool or smartphone application to better monitor and track patient activities and symptoms. Such monitoring tools have been developed for UC.^33-36^

The Global UC Narrative Advisory Panel surveys are the first to provide extensive worldwide data on communication between patients with UC and physicians; however, limitations exist. The interpretation of patient survey findings was limited by those patients who self-reported a diagnosis of UC, and by relying upon patients’ accurate recall of UC management and their understanding of survey questions. Furthermore, disease severity was determined by patient-reported medication history with no clinical assessment to determine disease activity. These surveys were fielded in 10 countries, but findings may not be applicable to all countries because of regional differences in the management of UC. Participant responses may have been influenced by cultural variations and differences between health systems/patient engagement with appointments and access to UC treatments. Although racial and ethnic disparities in health care quality have previously been reported,^37^ the total sample size in the patient survey was not large enough to enable subgroup analysis by race and ethnicity. Proportional weighting was used to generate global totals; data from smaller countries may thus have gotten weighted down, reducing their “share of voice” when all countries were grouped together. However, representative weighting is also a strength of the study because the findings may be expected to more accurately reflect the populations surveyed. The fact that patient and physician survey populations were independent of each other could be noted as a limitation (lack of direct relationship between patient and physician data) or a strength (identification of differences). Finally, survey questions were not formally validated but were designed to reflect patient and physician experiences and perceptions.

## CONCLUSIONS

Effective communication between physicians and their patients can help ensure that patients have a good knowledge and understanding of their disease and treatment options and can empower patients to seek other sources of information. Informed patients are more likely to engage with physicians in a shared decision-making process. Physicians should be aware of the gaps in patient-physician communication relating to quality of life, emotional concerns, and sexual/relationship concerns, as highlighted by this survey, and they should identify opportunities to address these issues during routine appointments. Physicians face a number of challenges for effective communication with patients: first, physicians need to address patient expectations and fears around an increasing number of UC treatment options; second, physicians need to be mindful of those topics of greatest concern to the patient and identify ways to discuss topics important to both parties, because shortage of appointment time is a barrier to communication; third, physicians have to satisfy the desire of their patients to be directed to sources of accurate information and emotional support; finally, physicians need to consider which of the available tools are most effective for helping patients to talk more openly and accurately about their disease and symptoms. Tackling these challenges can lead to improved patient-to-physician communication and implementation of a shared decision-making approach, thus enhancing patient experience and improving disease outcomes.

## Supplementary Material

izaa257_suppl_Supplementary_Material

## Notes

Author contributions: Study design: The Harris Poll, UC Narrative Survey Panel. Study support: GCI Health, The Harris Poll. Statistical analysis: The Harris Poll. Manuscript drafting, critical revision, and final approval: All authors.

Previous presentation: Parts of the data in this manuscript were previously presented at the Crohn’s and Colitis Congress (2019) and the 14th Congress of the European Crohn’s and Colitis Organisation (2019).

Supported by: The UC Narrative Surveys were sponsored by Pfizer Inc.

Conflicts of interest: David T. Rubin has received consultancy fees from AbbVie, AbGenomics, Allergan, Amgen, Celgene Corporation, Ferring Pharmaceuticals, Inc., Forward Pharma, Janssen Pharmaceuticals, Medtronic, Merck & Co., Inc., Napo Pharmaceuticals, Pfizer Inc, Salix Pharmaceuticals, Shire, Takeda, and TARGET PharmaSolutions. He has received financial support for research from AbbVie, Genentech/Roche, Janssen Pharmaceuticals, Prometheus Laboratories, Shire, and UCB.

Ailsa Hart has received consultancy fees from AbbVie, Allergan, Atlantic, Bristol-Myers Squibb, Celltrion, Falk, Ferring, Janssen, MSD, Napp Pharmaceuticals, Pfizer Inc., Pharmacosmos, Shire, and Takeda. She has received lecture fees from AbbVie, Atlantic, Bristol-Myers Squibb, Celltrion, Falk, Ferring, Janssen, MSD, Napp Pharmaceuticals, Pfizer Inc., Pharmacosmos, Shire, and Takeda. She has served as an advisory board member for AbbVie, Atlantic, Bristol-Myers Squibb, Celltrion, Falk, Ferring, Janssen, MSD, Napp Pharmaceuticals, Pfizer Inc., Pharmacosmos, Shire, Takeda, and the Genentech global steering committee.

Remo Panaccione has received consultancy fees from Abbott, AbbVie, Alba Therapeutics, Allergan, Amgen, Aptalis, AstraZeneca, Atlantic Healthcare, Baxter, Biogen Idec, Boehringer Ingelheim, Bristol-Myers Squibb, Celgene, Coronado Biosciences, Cosmo Technologies, Cubist, Eisai Medical Research, Elan, Eli Lilly, enGene, EnteroMedics, Exagen Diagnostics, Ferring, Genentech, Genzyme, Gilead, Given Imaging, GSK, Hospira, Human Genome Sciences, Janssen, Merck & Co., Merck Research Laboratories, Merck Serono, Millennium, Nisshin Kyorin, Novo Nordisk, Pfizer Inc, Qu Biologics, Receptos, Relypsa, Salient, Salix Pharmaceuticals, Santarus, Shire, Sigmoid Pharma, and Takeda. He has received speakers’ bureau fees from AbbVie, Aptalis, Celgene, Ferring, Janssen, Merck, Pfizer Inc, Prometheus Laboratories, Shire, and Takeda. He has received financial support for research from AbbVie, Ferring, Janssen, Shire, and Takeda.

Alessandro Armuzzi has received consultancy fees from AbbVie, Allergan, Amgen, Biogen Idec, Bristol-Myers Squibb, Celgene, Celltrion, Eli Lilly, Ferring, Hospira, Janssen, MSD, Mundipharma, Mylan, Pfizer Inc, Roche, Samsung Bioepis, Sandoz, Sofar, and Takeda. He has received financial support for research from MSD, Pfizer Inc, and Takeda. He has received lecture fees from AbbVie, Amgen, AstraZeneca, Biogen Idec, Bristol-Myers Squibb, Chiesi, Ferring, Hospira, Janssen, Mitsubishi-Tanabe, MSD, Mundipharma, Nikkiso, Otsuka, Pfizer Inc, Samsung Bioepis, Sandoz, Takeda, TiGenix, and Zambon.

Ulla Suvanto is an executive director of the Crohn and Colitis Association of Finland. Laura Wingate is Senior Vice President, Education, Support, & Advocacy for the Crohn’s and Colitis Foundation, New York, NY. J. Jasper Deuring_,_ John Woolcott, and Joseph C. Cappelleri are employees and stockholders of Pfizer Inc. Kathy Steinberg is an employee of The Harris Poll. Stefan Schreiber has received consultancy fees from AbbVie, Arena, Biogen, Bristol-Myers Squibb, Celgene, Celltrion, Falk, Fresenius, Gilead, IMAB, Janssen, MSD, Mylan, Pfizer Inc., Protagonist, Provention Bio, Takeda, and Theravance.

## References

[1] Ungaro R , Mehandru S, Allen PB, et al. Ulcerative colitis. Lancet. 2017;389:1756–1770. 27914657 10.1016/S0140-6736(16)32126-2 PMC6487890

[2] Ordás I , Eckmann L, Talamini M, et al. Ulcerative colitis. Lancet. 2012;380:1606–1619. 22914296 10.1016/S0140-6736(12)60150-0

[3] Solberg IC , Lygren I, Jahnsen J, et al.; IBSEN Study Group. Clinical course during the first 10 years of ulcerative colitis: results from a population-based inception cohort (IBSEN Study). Scand J Gastroenterol. 2009;44:431–440. 19101844 10.1080/00365520802600961

[4] Peyrin-Biroulet L , Panes J, Sandborn WJ, et al Defining disease severity in inflammatory bowel diseases: current and future directions. Clin Gastroenterol Hepatol. 2016;14:348–354.e17. 26071941 10.1016/j.cgh.2015.06.001

[5] Ghosh S , Mitchell R. Impact of inflammatory bowel disease on quality of life: results of the European Federation of Crohn’s and Ulcerative Colitis Associations (EFCCA) patient survey. J Crohns Colitis. 2007;1:10–20. 21172179 10.1016/j.crohns.2007.06.005

[6] Rubin DT , Dubinsky MC, Martino S, et al. Communication between physicians and patients with ulcerative colitis: reflections and insights from a qualitative study of in-office patient-physician visits. Inflamm Bowel Dis. 2017;23:494–501. 28296817 10.1097/MIB.0000000000001048 PMC5495553

[7] Rubin DT , Krugliak Cleveland N. Using a treat-to-target management strategy to improve the doctor-patient relationship in inflammatory bowel disease. Am J Gastroenterol. 2015;110:1252–1256. 25848924 10.1038/ajg.2015.86

[8] Baars JE , Siegel CA, Kuipers EJ, et al Patient’s perspectives important for early anti-tumor necrosis factor treatment in inflammatory bowel disease. Digestion. 2009;79:30–35. 19246918 10.1159/000203638 PMC2846412

[9] Glass KE , Wills CE, Holloman C, et al. Shared decision making and other variables as correlates of satisfaction with health care decisions in a United States national survey. Patient Educ Couns. 2012;88:100–105. 22410642 10.1016/j.pec.2012.02.010 PMC3492880

[10] Lai C , Sceats LA, Qiu W, et al. Patient decision-making in severe inflammatory bowel disease: the need for improved communication of treatment options and preferences. Colorectal Dis. 2019;21:1406–1414. 31295766 10.1111/codi.14759

[11] Rubin DT , Siegel CA, Kane SV, et al. Impact of ulcerative colitis from patients’ and physicians’ perspectives: results from the UC: NORMAL survey. Inflamm Bowel Dis. 2009;15:581–588. 19067414 10.1002/ibd.20793

[12] Schreiber S , Panés J, Louis E, et al. Perception gaps between patients with ulcerative colitis and healthcare professionals: an online survey. BMC Gastroenterol. 2012;12:108. 22894661 10.1186/1471-230X-12-108 PMC3523079

[13] Irving P , Burisch J, Driscoll R, et al. IBD2020 global forum: results of an international patient survey on quality of care. Intest Res. 2018;16:537–545. 30301341 10.5217/ir.2018.00041 PMC6223463

[14] Lönnfors S , Vermeire S, Greco M, et al. IBD and health-related quality of life—discovering the true impact. J Crohns Colitis. 2014;8:1281–1286. 24662394 10.1016/j.crohns.2014.03.005

[15] Afzali A , Armuzzi A, Bouhnik Y, et al Patient and physician perspectives on the management of inflammatory bowel disease: role of steroids in the context of biologic therapy. J Crohns Colitis. 2020;14:S366–S367.

[16] Physician Payments Sunshine Act of 2009, S 301, 111th Cong (2009). Accessed January 20, 2020. https://www.govtrack.us/congress/bills/111/s301.

[17] IBM Corp. IBM SPSS Statistics for Windows. Version 24.0. Armonk, NY: IBM Corp; 2016.

[18] Kish L Survey Sampling. New York, NY: John Wiley & Sons; 1965.

[19] Boeri M , Myers K, Ervin C, et al. Patient and physician preferences for ulcerative colitis treatments in the United States. Clin Exp Gastroenterol. 2019;12:263–278. 31354328 10.2147/CEG.S206970 PMC6572717

[20] Casellas F , Herrera-de Guise C, Robles V, et al. Patient preferences for inflammatory bowel disease treatment objectives. Dig Liver Dis. 2017;49:152–156. 27717791 10.1016/j.dld.2016.09.009

[21] Carpio D , López-Sanromán A, Calvet X, et al. Perception of disease burden and treatment satisfaction in patients with ulcerative colitis from outpatient clinics in Spain: UC-LIFE survey. Eur J Gastroenterol Hepatol. 2016;28:1056–1064. 27286569 10.1097/MEG.0000000000000658

[22] D’Incà R , Bertomoro P, Mazzocco K, et al. Risk factors for non-adherence to medication in inflammatory bowel disease patients. Aliment Pharmacol Ther. 2008;27:166–172. 17949472 10.1111/j.1365-2036.2007.03555.x

[23] Siegel CA . Shared decision making in inflammatory bowel disease: helping patients understand the tradeoffs between treatment options. Gut. 2012;61: 459–465. 22187072 10.1136/gutjnl-2011-300988

[24] Vaucher C , Maillard MH, Froehlich F, et al. Patients and gastroenterologists’ perceptions of treatments for inflammatory bowel diseases: do their perspectives match? Scand J Gastroenterol. 2016;51:1056–1061. 26891800 10.3109/00365521.2016.1147065

[25] López-Sanromán A , Carpio D, Calvet X, et al. Perceived emotional and psychological impact of ulcerative colitis on outpatients in Spain: UC-LIFE survey. Dig Dis Sci. 2017;62:207–216. 27817123 10.1007/s10620-016-4363-3

[26] Lerrigo R , Coffey JTR, Kravitz JL, et al The emotional toll of inflammatory bowel disease: using machine learning to analyze online community forum disclosure. Crohns Colitis 360. 2019;1:otz011.

[27] Coulson NS . How do online patient support communities affect the experience of inflammatory bowel disease? An online survey. JRSM Short Rep. 2013;4:2042533313478004. 24040493 10.1177/2042533313478004 PMC3767062

[28] Reich J , Guo L, Hall J, et al. A survey of social media use and preferences in patients with inflammatory bowel disease. Inflamm Bowel Dis. 2016;22:2678–2687. 27755269 10.1097/MIB.0000000000000951

[29] Pittet V , Vaucher C, Maillard MH, et al. Information needs and concerns of patients with inflammatory bowel disease: what can we learn from participants in a bilingual clinical cohort? PLoS One. 2016;11:e0150620. 26939069 10.1371/journal.pone.0150620 PMC4777374

[30] Argüelles-Arias F , Carpio D, Calvet X, et al. Knowledge of disease and access to a specialist reported by Spanish patients with ulcerative colitis. UC-LIFE survey. Rev Esp Enferm Dig. 2017;109:421–429. 28605920 10.17235/reed.2017.4748/2016

[31] Catalán-Serra I , Huguet-Malavés JM, Mínguez M, et al. Information resources used by patients with inflammatory bowel disease: satisfaction, expectations and information gaps. Gastroenterol Hepatol. 2015;38:355–363. 25813702 10.1016/j.gastrohep.2014.09.003

[32] Politi P , Bodini P, Mortilla MG, et al.; European Collaborative Study Group on Inflammatory Bowel Disease. Communication of information to patients with inflammatory bowel disease: a European Collaborative Study in a multinational prospective inception cohort. J Crohns Colitis. 2008;2:226–232. 21172215 10.1016/j.crohns.2008.01.007

[33] Walsh A , Matini L, Hinds C, et al. Real-time data monitoring for ulcerative colitis: patient perception and qualitative analysis. Intest Res. 2019;17:365–374. 31146510 10.5217/ir.2018.00173 PMC6667366

[34] Atreja A , Khan S, Rogers JD, et al.; HealthPROMISE Consortium Group. Impact of the Mobile HealthPROMISE platform on the quality of care and quality of life in patients with inflammatory bowel disease: study protocol of a pragmatic randomized controlled trial. JMIR Res Protoc. 2015;4:e23. 25693610 10.2196/resprot.4042 PMC4376196

[35] Elkjaer M , Shuhaibar M, Burisch J, et al. E-health empowers patients with ulcerative colitis: a randomised controlled trial of the web-guided “constant-care” approach. Gut. 2010;59:1652–1661. 21071584 10.1136/gut.2010.220160

[36] de Jong MJ , van der Meulen-de Jong AE, Romberg-Camps MJ, et al. Telemedicine for management of inflammatory bowel disease (myIBDcoach): a pragmatic, multicentre, randomised controlled trial. Lancet. 2017;390:959–968. 28716313 10.1016/S0140-6736(17)31327-2

[37] Fiscella K , Sanders MR. Racial and ethnic disparities in the quality of health care. Annu Rev Public Health. 2016;37:375–394. 26789384 10.1146/annurev-publhealth-032315-021439

