# Supplementary material for: Ulcerative Colitis Narrative Global Survey Findings: Communication Gaps and Agreements Between Patients and Physicians
Source: Inflamm Bowel Dis. 2020 Oct 15;27(7):1096–106. doi: 10.1093/ibd/izaa257 (PMC8214018; doi:10.1093/ibd/izaa257)
Supplement: izaa257_suppl_Supplementary_Material [file izaa257_suppl_supplementary_material.pdf]

SUPPLEMENTARY MATERIALS

SUPPLEMENTARY TABLE 1. Global response to the UC Narrative patient and physician surveys

**Country**	**Number of patient respondents**	**Patient recruitment target**	**Number of physician respondents**	**Physician recruitment target**
Australia	215	150	90	80
Canada	215	150	80	75
Finland	105	100	17	12
France	169	150	154	150
Germany	210	150	152	150
Italy	210	150	157	150
Japan	210	150	151	150
Spain	214	150	151	150
UK	251	150	153	150
US	301	150	149	150

UC, ulcerative colitis.

Supplementary materials 1. Patient Survey

Q264. In which country or region do you currently reside?

Q268. Are you...?

Q280. Respondent age.

Q364. What is your marital status?

Q700. How would you describe your current overall health?

Q600. Have you personally been told by a doctor that you have any of the following health conditions? Please select all that apply.

Q620. Have you seen a gastroenterologist/internist or another provider at a gastroenterologist/internist's office or clinic for your UC in the past 12 months? Please select all that apply.

Q615. Which healthcare professionals, if any, do you currently see to manage your UC? When thinking about managing your UC, please include all healthcare professionals involved in helping you live with and treat the symptoms of your UC, such as managing your medication, treating the inflammation of your colon, etc. Please select all that apply.

Q625. Please indicate all prescription medications you have ever taken for UC. Please select all that apply.

Q630. Please indicate all prescription medications you are currently taking for UC.

Q635. You indicated that you have taken a corticosteroid for your UC. In the past 12 months, for approximately how many months have or had you taken corticosteroids for your UC? If you have taken corticosteroids for 1 or more days within a 30-day period, please count as one month.

Q636. You indicated that you are currently taking a biologic for your UC. Is the healthcare provider who prescribes your biologic treatment the provider who is mostly responsible for managing your UC?

Q398. Next, we have a few employment and economic questions.

1. Are you employed full time for pay with an organization or company?
2. Are you employed part time for pay with an organization or company?
3. Are you self-employed full time?
4. Are you self-employed part time?

Q402. Which of the following best describes your current situation?

Q404. Do any of the following describe you? Please select all that apply.

Q410. Which one of the following best describes your employment status?

Q1305. How many children are you the parent or guardian of?

Q601. To the best of your knowledge, do you have a family history of inflammatory bowel disease (IBD), such as Crohn’s disease or ulcerative colitis (UC)?

Q606. How old were you when you first experienced UC symptoms? Your best estimate is fine.

Q607. How old were you when you were first diagnosed with UC by a doctor? Your best estimate is fine.

Q705. How many times do you go to the bathroom for any reason, including to pass stool, air, blood, or mucus, on your best day? Please think of a 24-hour period. Please do not include trips to the bathroom only to urinate. Your best estimate is fine.

Q710. How many times do you go to the bathroom for any reason, including to pass stool, air, blood, or mucus, on your worst day? Please think of a 24-hour period. Please do not include trips to the bathroom only to urinate. Your best estimate is fine.

Q715. What is important to you in managing your UC? Please select all that apply.

Q720. In general, when thinking about managing your UC, which of the following, if any, are your top 3 worries because of your UC?

Q725. How much do you agree or disagree with each of the following statements about the overall impact of UC on your life?

1. I often feel like I spend more time in the bathroom than anywhere else.
2. I feel like I would be a more successful person if I did not have UC.
3. I worry I will pass my UC to my (future) children.
4. UC has made me more appreciative of the important things in life.
5. UC has made me more resilient.
6. I feel comfortable discussing my health issues with my family/friends.
7. UC is mentally exhausting.
8. I feel that UC controls my life, rather than me controlling the disease.

Q805. Which of the following emotions, if any, do you typically experience during a UC flare?

Q816. Do you currently consider your UC to be in remission? When we say remission, we mean that your disease is controlled with few to no symptoms.

Q810. How many separate UC flares have you had in the past 12 months? If you are not sure, please provide your best estimate. When we say flare, we mean a period of time where you experience a dramatic increase in symptoms that is different than what you typically experience.

Q815. Thinking of your most recent UC flare, how long did the flare last? If you are not sure, please provide your best estimate.

Q900. In the past 12 months, about how many of the following events, if any, have you missed due to your UC? Your best estimate is fine.

1. My child’s events (e.g. parent-teacher conferences, sporting events, plays, etc.).
2. Days of work due to disease/symptoms.
3. Days of work due to treatment or medical appointments (e.g. infusion appointments).

4. Days of school due to disease/symptoms.

5. Days of school due to treatment or medical appointments (e.g. infusion appointments).

6. Social events (e.g. weddings, parties, bat/bar mitzvah, dinner with friends, movies, etc.) [Exact events differed by country].

7. Travel plans (work or pleasure).

Q905. Have you done any of the following as a result of your UC? Please select all that apply.

Q910. Have you done any of the following as a result of your UC? Please select all that apply.

Q915. How much do you agree or disagree with each of the following statements about the impact of UC on work?

Q915_1. How much do you agree or disagree with each of the following statements about the impact of UC on work?

1. My UC has had a negative effect on my confidence at work.

2. I am too tired because of my UC to excel in my workplace.

3. I feel comfortable discussing my health issues in my workplace.

4. My employer is very understanding of my condition.

5. I am in too much pain or distracted by the daily needs of my disease to focus on my work.

6. I have not told my employer about my UC because I fear the repercussions.

7. Managing my UC has made me better at managing my workload.

Q1000. Have you ever...?

Q1001. How much do you agree or disagree with the following statement? I wish I knew about patient associations or organizations earlier.

Q1002. What information, if any, would you be interested in receiving from a patient association or organization?

Q1047. Earlier, you mentioned that you currently see multiple healthcare providers to manage your UC. Who do you primarily communicate with about each of the following aspects of your UC?

Q1047. Earlier, you mentioned that you currently see multiple healthcare providers to manage your UC. Who do you primarily communicate with about each of the following aspects of your UC?

1. Emotional impacts.
2. Symptoms.
3. Side effects of treatment.
4. Treatment.
5. Your quality of life.

Q1010. Have you set goals for managing your UC with your gastroenterologist/internist? When we say goals, please think of what is important to you in managing your UC.

Q621. In the past 12 months, how many times have you visited your gastroenterologist/internist’s office for your UC? Your best estimate is fine.

Q622. Is the gastroenterologist/internist’s office you have visited in the past 12 months located in an inflammatory bowel disease (IBD) center/clinic?

Q623. Does the gastroenterologist/internist’s office you have visited in the past 12 months have inflammatory bowel disease (IBD) specialized nurse(s)?

Q1015. How satisfied are you with the communication you have with your gastroenterologist/internist regarding your UC?

Q1020. How satisfied are you with each of the following aspects for how you are managing your UC with your gastroenterologist/internist?

1. Discussion of how my current medication may help reach my long-term treatment goals.

2. Discussion of how my current medication may help reach my day-to-day goals (e.g. go out with friends).

3. Discussion of all my prescription medication options, including benefits and side effects.

4. Discussion of the possibility that my disease can be controlled with few to no symptoms.

5. Information about upcoming procedures (e.g. day-of schedule, recovery time, any long-term side effects, etc.).

6. Expectations set around my medication regimen (e.g. how to know if it is working, signs a medication change is necessary, etc.).

7. Willingness to discuss alternative therapies (e.g. acupuncture, natural remedies, etc.) despite lack of medical evidence to support efficacy [Not asked in Spain].

8. Discussion of mental/emotional health impacts of UC.

9. Expectations set around the long-term realities of living with a chronic and unpredictable disease.

10. Discussion of symptoms experienced.

11. Discussion of how treatment modality (i.e. injection, infusion, oral) impacts my quality of life.

12. Discussion of how I can get more support and information on my condition.

Q1025. How well do you feel that your gastroenterologist/internist has explained each of the following aspects of managing your UC to you?

1. Lifestyle changes (e.g. diet, exercise).

2. My current UC status/whether my UC has become better or worse.

3. Potential risk factors for cancer.

4. The benefits and risks of biologics.

5. How inflammation relates to my UC.

6. The importance of getting my inflammation under control, regardless of current symptoms.

7. What will happen if my UC treatment fails?

8. The hereditary nature of UC.

9. The importance of keeping up to date on health maintenance (e.g. vaccinations, examinations, colon cancer surveillance, etc.).

10. How to access information and support from patient associations or organizations.

Q1030. Which of the following topics related to your UC, if any, do you feel are the most important to prioritize during a routine appointment with your gastroenterologist/internist? Please select up to three.

Q1035. With respect to your communication with the gastroenterologist/internist mostly responsible for managing your UC, how much do you agree or disagree with each of the following statements?

1. I often regret not telling my gastroenterologist/internist more during my visits.

2. I wish my gastroenterologist/internist had discussed all available treatment options earlier, so I had a better idea of my choices.

3. My gastroenterologist/internist rarely has time to address all of my questions and concerns.

4. I wish my gastroenterologist/internist and I talked more about my goals for managing my UC.

5. I feel comfortable raising concerns and fears with my gastroenterologist/internist.

6. I don’t feel comfortable talking to my gastroenterologist/internist about emotional concerns.

7. I don’t feel comfortable talking to my gastroenterologist/internist about my sex life and personal relationship concerns.

8. I wish my gastroenterologist/internist and I talked more about my fears of medical treatments.

9. I am hesitant to tell my gastroenterologist/internist if I don’t take my medication exactly as prescribed.

10. I am honest with my gastroenterologist/internist when discussing my experiences with UC.

11. I wish I had more time at appointments with my gastroenterologist/internist.

Q1040. Which of the following, if any, do you wish your gastroenterologist/internist better understood about your experiences living with UC? Please select all that apply.

Q1045. With respect to your communication with the gastroenterologist/internist mostly responsible for managing your UC, how much do you agree or disagree with each of the following statements?

1. When I leave an appointment with my gastroenterologist/internist, I know exactly how to follow his/her treatment recommendations.

2. I worry that, if I ask too many questions, my gastroenterologist/internist will see me as a difficult patient and it will affect the quality of care I receive.

3. I feel that my gastroenterologist/internist is prescribing the very best available medication for my unique set of symptoms and lifestyle issues.

4. Other healthcare providers in my gastroenterologist/internist’s office (e.g. nurses, physician assistants, etc.) play a strong role in educating me on treatments and lifestyle adjustments.

5. My gastroenterologist/internist and I work together to make decisions about my UC treatment plan.

Q1100. How many times, if any, have you been hospitalized for your UC in the past 12 months? When thinking of hospitalizations, please think of all times you have been to the hospital, including trips to the emergency room, being admitted to the hospital, etc.

Q1105. How satisfied are you with your current UC medications?

Q1110. For which of the following reasons are you satisfied?

Q1115. You mentioned you are not satisfied with your current UC medications.

For which of the following reasons are you not satisfied? Please select all that apply.

Q1120. How much do you agree or disagree with each of the following statements?

1. I wish I had more medication choices to treat my UC.

2. I wish I had moved to biologics sooner than I did.

3. I am/you are not happy with biologics.

4. I believe the benefit of biologics (medication taken through injection or infusion) outweighs the risks (e.g. side effects).

5. If my treatment makes me feel good enough, I don’t see a need to consider other treatment options, even if they might make me feel even better.

6. It is possible for a UC medication to give me back my old life (i.e. before I was diagnosed with UC).

7. I am/you are afraid if I go off steroids, I will immediately have a UC flare.

8. I wish I knew more about all the available medications for UC when I was first diagnosed.

9. I wish I knew where to find information and support when I was first diagnosed with UC.

10. Patient associations or organizations are important to the management of my UC.

Q1125. Which of the following methods of medication administration would you prefer, assuming they were all equally effective? Please only select one.

Q1130. As far as you know, are the following statements about UC treatment true or false, or are you not at all sure?

1. If my symptoms are under control, there is not active disease or inflammation.
2. It is important to keep my disease under control to reduce long-term complications.
3. Uncontrolled inflammation is a risk factor for colorectal cancer.
4. UC may be associated with other conditions outside of my colon.
5. It is okay to stop taking UC medications once I feel better.
6. It is okay to use steroids as a long-term maintenance medication.
7. It is possible for my body to stop responding to biologics, causing the medication to no longer work.

Q1130. Knowledge Score Card – number of responses correct

Q1205. When thinking about your UC medication(s), what are the top three indicators that it is time to consider changing or adding medications? Please select up to three.

Q1220. For which of the following reasons, if any, have you been reluctant when your gastroenterologist/internist has recommended or made a change in your medication regimen? Please select all that apply.

Supplementary materials 2. Physician Questionnaire

Q8625. What is your primary medical specialty?

Q8732. In what country is your practice located? If you practice in more than one country, please select the primary country in which you practice.

Q628. How many years have you been in your specialty practice?

Q8716. Which of the following best describes your medical practice?

Q655. Do you currently practice in an IBD center/clinic?

Q656. Does your primary medical practice have IBD specialized nurse(s)?

Q657. Does your practice offer patients multidisciplinary care at one location?

Q630. Approximately how many unique adult patients (ages 18 and older) do you see in your practice each month who have the following conditions? For each condition, please think about all of the patients you see or treat per month. If you are not sure, please provide your best estimate.

1. Ulcerative colitis
2. Irritable bowel syndrome
3. Crohn’s disease
4. Celiac disease

Q645_1. Approximately what proportion of your ulcerative colitis (UC) patients are currently being treated by the following types of prescription medications? Your best estimate is fine. As patients may be on multiple therapies for their UC, the sum of these proportions may exceed 100%.

1. Immunosuppressant.
2. Anti-TNF biologic.
3. Other biologic.
4. 5-ASA.

5. Corticosteroid.

6. Other.

7. Not currently taking prescription medication.

Q646. Do you personally initiate biologic treatment in your ulcerative colitis patients?

Q701. Approximately what proportion of your patients with moderate to severe UC are in the following age groups? Your best estimate is fine.

1. under age 18.

2. Age 18–24.

3. Age 25–34.

4. Age 35–44.

5. Age 45+.

6. Age 45–64.

7. Age 65+.

Q705. Based on what your patients tell you, what is important in managing their moderate to severe UC? Please select all that apply.

Q710. Which of the following aspects, if any, do you discuss with your patients with moderate to severe UC? Please select all that apply.

Q715. When do you typically discuss each of the following with your patients with moderate to severe UC?

1. The impact of UC on patients' job/career/education or ability to attend work/school.

2. The impact of UC on patients' mental/emotional health.

3. The impact of UC on patients' ability to conceive (i.e. get pregnant)/father a child.

4. Whether or not patients seek treatment from other healthcare professionals (e.g. primary care physician, physician assistant, internist, psychologist, nurse [ex-Japan: /nurse practitioner], pharmacist, dietitian etc.).

5. Patients’ ability to afford their medication(s).

6. Patients’ ability to adhere to their prescribed medication regimen.

7. Patients’ preference on medication type.

8. The impact of UC on patients’ sex lives and personal relationships.

9. Patients’ ability to manage flares.

10. Whether or not the patient has adequate support from family and friends.

11. Patients’ lifestyle goals for managing their UC (e.g. participate in a hobby, be able to travel).

12. Whether or not patients are interested in or should seek out complementary alternative treatments.

13. The difficulties my patients have in communicating aspects of their disease to those around them.

14. The benefits and risks of treatment options.

15. Patients’ concerns about medication side effects.

16. The information and support patient associations or organizations can provide.

Q715. When do you typically discuss each of the following with your patients with moderate to severe UC?

1. The impact of UC on patients’ job/career/education or ability to attend work/school.

2. The impact of UC on patients’ mental/emotional health.

3. The impact of UC on patients’ ability to conceive (i.e. get pregnant)/father a child.

4. Whether or not patients seek treatment from other healthcare professionals (e.g. primary care physician, physician assistant, internist, psychologist, nurse [ex-Japan: /nurse practitioner], pharmacist, dietitian etc.).

5. Patients’ ability to afford their medication(s).

6. Patients’ ability to adhere to their prescribed medication regimen.

7. Patients' preference on medication type.

8. The impact of UC on patients' sex lives and personal relationships.

9. Patients' ability to manage flares.

10. Whether or not the patient has adequate support from family and friends.

11. Patients' lifestyle goals for managing their UC (e.g. participate in a hobby, be able to travel).

12. Whether or not patients are interested in or should seek out complementary alternative treatments.

13. The difficulties my patients have in communicating aspects of their disease to those around them.

14. The benefits and risks of treatment options.

15. Patients' concerns about medication side effects.

16. The information and support patient associations or organizations can provide.

Q720. Based on your conversations with your patients with moderate to severe UC, which of the following, if any, are your patients' top three worries because of their UC? Please select up to three.

Q725. With respect to your communication with your patients with moderate to severe UC, how much do you agree or disagree with each of the following statements?

1. I wish my UC patients and I talked more about their UC treatment goals.

2. My patients understand their disease and the treatment options available.

3. I wish my patients understood the damage that long-term inflammation can have.

4. My patients are honest with me when discussing their experiences with UC.

5. I have taken steps to improve my communication skills with my patients (e.g. attended seminars, taken training courses, etc.).

6. I wish there was a way my patients could communicate with me more frequently while experiencing symptoms in between visits.

7. I wish my patients and I talked more about their fears of medical treatments.

8. I wish I had more time at appointments with my UC patients.

9. I wish I had access to an IBD nurse to help me manage UC patients at my practice.

Q800. What proportion of your patients with moderate to severe UC do you recommend patient associations or organizations to as a source for information and support? Your best estimate is fine.

Q805. How well do you think your patients with moderate to severe UC understand each of the following aspects of UC?

1. Even if their UC symptoms are under control, they can still have active disease or inflammation.

2. It is important to keep their disease under control to reduce long-term complications.

3. Uncontrolled inflammation is a risk factor for colorectal cancer.

4. UC may be associated with other conditions outside their colon.

5. It is important to continue taking UC medications, even if they are feeling better.

6. Steroids should not be used as a long-term maintenance medication.

7. It is possible for their body to stop responding to biologics, causing the medication to no longer work.

Q1000. How much do you agree or disagree with each of the following statements?

1. UC patients who are involved in making treatment decisions tend to be more satisfied with their treatment experience than those who are not as involved.

2. UC patients often settle for a treatment that makes them feel ‘good enough’ even though their disease is active or not well-controlled.

3. I wish my patients would talk to me before they stopped their medications.

4. I wish I had more time to discuss all available treatments earlier, so my patients had a better idea of their choices.

5. I wish I moved more than half of my patients who are currently taking biologics to biologic therapy earlier than I did. (Prescribe biologics [all US respondents]).

6. More than half of my current biologic patients wish they had moved to biologic therapy sooner than they did. (Prescribe biologics [all US respondents]).

7. My patients are comfortable being on steroids as long as they feel better.

8. More than half of my patients take their prescriptions exactly as prescribed.

Q1002. How much do you agree or disagree with each of the following statements?

Please continue to think about your experiences treating patients in your practice who have moderate to severe UC.

1. I rarely have time to address all of my UC patients' questions and concerns.

2. My patients feel comfortable raising concerns and fears with me.

3. I spend time discussing my patients' diet with them.

4. My patients feel comfortable talking to me about their emotional concerns.

5. My patients don't feel comfortable talking to me about their sex lives and personal relationship concerns.

6. Patient associations or organizations are important to the management of UC.

7. I wish there were resources I could refer my patients to for information and support.

8. More than half of my patients have requested cheaper medication alternatives.

9. My patients would prefer an effective oral medication over an injectable one if given the choice.

Q1005. How much do you agree or disagree with each of the following statements?

1. More than half of my UC patients believe that pain and cramping are just part of living with UC.

2. More than half of my UC patients believe that spending significant time in the bathroom is just part of living with UC.
3. More than half of my UC patients have accepted that having UC means that they have to settle for a reduced quality of life.
4. My patients would approach their career or education differently if they did not have UC.
5. My patients would approach their personal relationships differently if they did not have UC.
6. More than half of my UC patients believe that urgency in going to the bathroom is just part of living with UC.

Q1010. What proportion of your patients with moderate to severe UC do you feel are satisfied with the communication they have with you regarding their UC? Your best estimate is fine.

1. Very dissatisfied.
2. Somewhat dissatisfied.
3. Somewhat satisfied.
4. Very satisfied.

Q1015. Which of the following topics, if any, do you feel are the most important to prioritize during a routine appointment with your patients with moderate to severe UC? Please select up to three.

Q1020. In thinking about your patients with moderate to severe UC, which of the following, if any, would help to improve your patient relationships? Please select all that apply.

Q1100. Turning your attention to medication specifically, what proportion of your patients with moderate to severe UC do you feel are satisfied with their current medications? Your best estimate is fine.

1. Very dissatisfied.
2. Somewhat dissatisfied.
3. Somewhat satisfied.
4. Very satisfied.

Q1105. Which of the following do you typically do to determine if your patients are satisfied with their medication regimen? Please select all that apply.

Q1110. Approximately what proportion of your patients with moderate to severe UC do you feel are well controlled with few to no symptoms by their current UC medication regimen? Please provide your best estimate.

Q1200. What are the top three things patients tell you that indicate to you that it is time to consider changing their medication(s)? Please select up to three.

Q1210. For which of the following reasons, if any, have your patients with moderate to severe UC been reluctant when you recommend or make a change in their medication regimen? Please select all that apply.

Q1215. To what extent, if any, would each of the following make you hesitate to prescribe patients with moderate to severe UC an anti-TNF biologic therapy? (Examples varied by country).

1. Concerns about the side effects of these medications.
2. Fear of the potential long-term risks.
3. Feeling the patient is doing well enough on their current medication.
4. Feeling the patient is controlled on their current medication.
5. Patients may decline to move to anti-TNF biologic therapy.
6. I would want more information about the medication before initiating new treatment.
7. The possible financial burden on the patient.
8. Not covered by their healthcare plan/insurance.

9. The impact of UC on patients’ ability to conceive (i.e. get pregnant)/father a child.

Q1220. To what extent, if any, would each of the following make you hesitate to prescribe patients with moderate to severe UC an anti-integrin biologic therapy? (Examples varied by country).

1. Concerns about the side effects of these medications.
2. Fear of the potential long-term risks.
3. Feeling the patient is doing well enough on their current medication.
4. Feeling the patient is controlled on their current medication.
5. Patients may decline to move to anti-integrin biologic therapy.
6. I would want more information about the medication before initiating new treatment.
7. The possible financial burden on the patient.
8. Not covered by their healthcare plan/insurance.
9. The impact of UC on patients’ ability to conceive (i.e. get pregnant)/father a child.

Q1222. Earlier you mentioned that you do not personally initiate biologic treatment for your UC patients. Do you ever discuss biologic treatment with your UC patients?

Q1300. Have you completed a fellowship in IBD?

Q8702. Are you...?

Q8705. Age

Q8710. In what year did you graduate from medical school?

Q8712. In what year did you complete your residency?

Q8719. How would you describe your office or clinic?

Q8722. On average, how many patients do you see in a typical week? If you are not sure, your best estimate will do.

Q8725. Which of the following best describes the ages of your patient population?

Q8728. On average, about how many prescriptions do you write (or medications do you dispense) in a week? If you are not sure, your best estimate will do.

Supplementary materials 3. Patient and Physician Recruitment

3.1 Patient Recruitment

Patients with UC were recruited from online panels of adults who had agreed to participate in market research, and were subsequently screened for qualification based on the criteria for the UC Narrative Survey. For each country, a pre-determined survey respondent target was set (Supplementary Table 1), and once achieved the survey was closed.

Recruitment for Online Panels

Members of such online panels are recruited from a multitude of both online and offline sources, including but not limited to: co-registration offers on partners' websites; targeted emails sent by online partners to their audiences; graphical and text banner placement on partners' websites (including social media, news, search, and community portals); trade show presentations; targeted postal mail invitations; and telephone recruitment of targeted populations.

Each recruitment source is carefully vetted through a rigorous interviewing and testing process and then monitored for response quality on an ongoing basis. Each database of respondent information is actively screened and updated along numerous demographic and psychographic variables to allow for precision and quality in the online sample we provide.

Recruitment/Screening for Patients with UC

Email invites were sent to a representative mix of adults (members of online panels) in each country, and participants who clicked on the link were taken through a series of screener questions for verification and qualification (as outlined in the methods section of the manuscript).

Due to the various ways in which respondents were recruited across countries, neither the total number of respondents recruited nor the actual response rate achieved could be calculated.

3.2 Physician Recruitment

Physicians were recruited online from a database of physicians collated through various channels such as CME activities, clinical websites, peer-reviewed communications, physician social media platforms, and market research. Each member of the database was verified via automated and manual processes. Physicians were also recruited via telephone using an existing physician database, and desk research such as directories and websites of associations/physicians. For each country, a pre-determined survey respondent target was set (Supplementary Table 1), and once achieved the survey was closed.

3.3 Compensation

Both qualifying patients and physicians were offered an incentive for completing the research.
